# Driving by frequent cannabis users ‘the morning after’ last use of smoked cannabis: an observational driving simulator study

**DOI:** 10.1186/s42238-026-00416-w

**Published:** 2026-03-03

**Authors:** Christina Zakala, Sampson Zhao, Alex Battistuzzi, Adrien Nette, Justin Matheson, Bernard Le Foll, Bruna Brands, Christine M. Wickens, Pamela Kaduri, Omer Hasan, Wei Wang, Sheng Chen, Patricia Di Ciano

**Affiliations:** 1 Institute for Mental Health Policy Research, Toronto, Canada; 2 Department of Pharmacology and Toxicology, University of Toronto, Toronto, Canada; 3 Trent University, Peterborough, Canada; 4 Addictions Division, Centre for Addiction and Mental Health, Toronto, Canada; 5 Department of Psychiatry, University of Toronto, Toronto, Canada; 6 Translational Addiction Research Laboratory, Centre for Addiction and Mental Health, Toronto, Canada; 7 Department of Family and Community Medicine, University of Toronto, Toronto, Canada; 8 Waypoint Center for Mental Health Care, Penetanguishene, Canada; 9 Campbell Family Mental Health Research Institute, Toronto, Canada 250 College Street, M5T 1R8; 10 Health Canada, Ottawa, Canada; 11 Dalla Lana School of Public Health, University of Toronto, Toronto, Canada; 12 Institute of Health Policy, Management and Evaluation, University of Toronto, Toronto, Canada; 13 Department of Psychiatry and Mental Health, Muhimbili University of Health and Allied Sciences, Dar es Salaam, Tanzania; 14 Biostatistics Core, Centre for Addiction and Mental Health, Toronto, Canada

**Keywords:** THC, CBD, Speed, Reaction time, Standard deviation of lateral position

## Abstract

**Background:**

It is well-established that cannabis can affect driving in the hours after cannabis use, but the exact duration of these effects, and relationship with delta-9-tetrahydrocannabinol (THC) concentrations in blood and oral fluid, remains to be determined.

**Methods:**

Frequent (≥ 4 times a week) users of smoked cannabis drove a simulator the morning after (12-15 hours) last use of smoked cannabis; a control group of non-cannabis users matched for age and sex was also included. Concentrations of THC, cannabidiol (CBD) and metabolites were measured in oral fluid and blood at the time of the drive.

**Results:**

In total, 65 participants (mean age 30 years; 33 males) in each group completed all study procedures. Participants were generally well-matched (age, sex, driving experience, amount of driving per year/week, hours of sleep) but differed in racial breakdown and years of education. Under both standard and dual task (distacted) conditions, standard deviation of lateral position (SDLP) was higher in the control group (standard: 0.305 meters; dual task: 0.272 meters; *n*=65) compared to the cannabis group (standard: 0.28 meters; dual task: 0.256 meters; *n*=65); these differences were small (Cohen’s d -0.389 (standard) and -0.359 (dual task)) and were not significant after correction for multiple comparisons. Measures of speed and following distance were not impacted. Neither blood nor oral fluid THC, CBD or metabolites was significantly correlated with any measure of driving after correction for multiple comparisons; mean concentrations of blood THC was above 2 ng/mL. After correction for multiple comparisons, trends between driving and concentrations of the psychoactive metabolite 11-hydroxy-THC (11-OH-THC) were found. Participants who smoked cannabis the night before reported higher levels of subjective intoxication, and more willingness to drive before the drive, that was not significant after correction for multiple comparisons.

**Conclusions:**

The regular cannabis use group showed no significant impairment in driving performance 12-15 hours after last cannabis use the night before, compared to the control group. Blood and oral fluid THC concentrations may not be an accurate correlate of driving behavior. Large-scale studies are needed to determine whether less frequent users are impaired the morning after last use, and whether the present findings also extend to different routes of administration.

## Introduction

With liberalization of cannabis legislation, there has been an increase in the prevalence of cannabis use (Hammond et al. 2023, Canada 2024), with more people reporting frequent use of cannabis in Canada (Hammond et al. 2023, Canada 2024), where cannabis has been legal since 2018; this has been associated with an increase in driving after use of cannabis (Kucera and Hammond 2025). Epidemiological studies have demonstrated that cannabis can increase the risk of a motor vehicle collision (Hostiuc et al. 2018, Brubacher et al. 2022, Jin et al. 2025, Preuss et al. 2021). Although the findings of laboratory studies can differ, a few recent reviews and systematic reviews demonstrated that cannabis use can increase ‘weaving’, slow reaction time, and produce compensatory decreases in speed and following distance (Ward and Dye 1999, Alvarez et al. 2021, Brands et al. 2021). The effects of cannabis are most pronounced in the hours after use (< 6 h) (Arkell et al. 2020). The evidence for residual effects of cannabis is mixed (McCartney et al. 2023, Brands et al. 2019); some studies have found that frequent users of cannabis for medical or non-medical purposes exhibit decrements in performance the day after last use of cannabis (Doroudgar et al. 2018, Dahlgren et al. 2020, Hartley et al. 2019), whereas some studies did not find residual effects (Brands et al. 2019, Mastropietro et al. 2025, Ronen et al. 2010, Ronen et al. 2008).

Deterrence of impaired driving depends on a combination of behavioral tests and biomarkers. If a police officer suspects a driver of being impaired by drugs, the driver can be pulled over and asked to complete the standardized field sobriety test (SFST; a battery of behavioral tests), and an oral fluid sample may also be taken at the roadside to detect one of three drugs, including delta-9-tetrahydrocannabinol (THC), the psychoactive component of cannabis. If, following the SFST and/or oral fluid test, a suspicion of driving after the use of drugs such as cannabis persists, further evaluation may include a sampling of blood to quantify THC. The legal threshold for THC in saliva and blood varies (Gjerde and Strand 2023). Some jurisdictions have a zero tolerance for THC in blood, while others have adopted per se thresholds in blood that are generally in the range of 2 or 5 ng/mL (Gjerde and Strand 2023).

At present, there is an uncertain relationship of blood and oral fluid THC to driving. Although it is well-established that there is a dose-response relationship of the dose of cannabis used to changes in driving (Ronen et al. 2008, Robbe 1998, Ramaekers and Robbe 2000, Lenne et al. 2010, Downey et al. 2013), there is less evidence for a straightforward linear relationship of blood THC to changes in driving. Recent studies have cast doubt on the possibility that blood THC is correlated with driving (Fitzgerald et al. 2023, Marcotte et al. 2022), and our recent systematic review found that only two of twelve studies found a linear correlation of blood THC to driving (Behzad et al. 2025). Despite this, there may be a threshold of blood THC above which driving is impaired (Di Ciano et al. 2023). Indeed, in studies of the residual effects of cannabis on driving it was found that participants with residual concentrations of THC greater than 2 or 5 ng/ml exhibited lower speed and poorer car following (Doroudgar et al. 2018). There is some evidence that oral fluid may provide a more sensitive measure of impairment than blood (Fitzgerald et al. 2023). However, few studies have been conducted to investigate the relationship of oral fluid THC to driving, and none have studied the possibility of a relationship between oral fluid THC and residual effects of cannabis on driving.

The purpose of the present study was to determine whether any ‘next day’ effects of cannabis can be observed in the 12–15 h after last use of smoked cannabis the night before. Frequent users were compared to participants who have not recently used cannabis. Frequent users were recruited because two studies have found evidence for residual effects of cannabis in these cannabis users (Doroudgar et al. 2018, Dahlgren et al. 2020). Given the uncertain relationship between blood THC and driving, blood was collected to determine whether there are any relationships between THC and driving. Further, a possible linear relationship of oral fluid THC to driving was investigated. We hypothesized that that there would be residual driving impairment in the cannabis group and that a relationship of driving to blood and/or oral fluid THC would be apparent. Although there is conflicting evidence about whether cannabis produces residual effects on driving performance, the present hypotheses were chosen because some previous papers found residual impairment in cannabis users (Doroudgar et al. 2018, Dahlgren et al. 2020, Hartley et al. 2019) and there were some relationships to concentrations of blood THC (Doroudgar et al. 2018).

## Methods

### Study design and procedures

This was a parallel group, open-label, single session, observational study of the effects of smoked cannabis on simulated driving and on blood and oral fluid THC concentrations ‘the morning after’ last use of smoked cannabis. A group of participants who frequently smoke cannabis was compared to a control group of participants who have not recently used cannabis. This study was conducted at the Centre for Addiction and Mental Health (CAMH) in Toronto, Canada. It was approved by the CAMH Research Ethics Board (#091/2024) and the Health Canada Research Ethics Board (2024–010 H). Participants were recruited between January and August, 2025. This study included a total of two sessions: one eligibility session and one test session. This manuscript conforms to the STROBE reporting guidelines (Cuschieri 2019) for observational studies, cohort designs.

### Participants

Inclusion criteria were (1) Aged 19–45 years (the lower limit is the legal age to consume cannabis in Ontario; the upper age limit was to control for the effects of age on cognition); (2) Abstain from alcohol and other drugs (apart from non-psychoactive prescribed medications) for 48 h prior to the test session; (3) A valid G or G2 driver’s licence (standard driver’s licence in Ontario) or equivalent from another jurisdiction; (4) No psychiatric comorbidities as determined by self-report; (5) No use of psychoactive medications or drugs including opioids for pain; (6) No current or past alcohol or other drug dependence, or problematic substance/drug use (self-report); (7) No participation in past driving studies at CAMH (to control for practice effects); (8) Breath alcohol reading of 0.0% on the test day; (9) Not pregnant or breastfeeding; (10) Any other reason to exclude, as judged by the study physician, if participation in the study is deemed to be unsafe; 11) Willing and able to provide informed consent. Additional inclusion criteria for the frequent cannabis users were as follows: (1) Currently smoke cannabis on average 4–7 days per week (only people who primarily smoked cannabis were recruited; the original inclusion criterion was smoke cannabis 5–7 days per week but this was too restrictive and the inclusion criterion was relaxed to facilitate recruitment); (2) Willing to smoke cannabis at home 12–15 h before the test session; (3) Willing to smoke cannabis in the form of a new commercially available, legal source pre-roll, and to bring the remnant of the ‘joint’ to the lab for weighing and its packaging to verify composition of the cannabis; (4) Use cannabis for non-medical purposes (to prevent the confound of co-morbidities). An additional inclusion criterion for the control group was not using cannabis in any form in the past month. Participants in the two groups were matched for age and sex.

### Recruitment

Participants were recruited through ads placed on local transit and by contacting people who had expressed interest in previous studies and consented to being contacted. Participants completed an initial screen over Research Electronic Data Capture (REDCap) (Harris et al. 2009), and those who met preliminary eligibility were invited to the lab for a full eligibility assessment.

### Eligibility session

Following an in-person consent, participants provided urine for a 14-panel screen (CLIAwaved) for determination of use of any psychoactive medications, and a breathalyzer test (Alert J5™; Alcohol Countermeasure Systems) was also conducted to verify no use of alcohol. Age and driver’s licence status were verified. Females in the cannabis group underwent a pregnancy test. The pattern of cannabis use over the past month was recorded with use of the Timeline Follow Back (Sobell et al. 1992). A brief medical history assessment was conducted. Concomitant medications were also recorded. Participants also drove the simulator during this session to ensure that they were comfortable with the procedures.

### Test session

For the cannabis group, the test session was scheduled 12–15 h after last use of smoked cannabis. Attempts were also made to schedule control group participants in the morning (to control for any possible effects of time of day). The time that the ‘joint’ was smoked was recorded. The composition of the cannabis, as indicated on the packaging, was recorded.

For all participants, prior to the test session, a breathalyzer (Alert™ J5; Alcohol Countermeasure Systems) was used. In addition, a 14-panel urine screen was used to confirm that there was no recent use of other recreational/psychoactive drugs (amphetamines, barbiturates, buprenorphine, benzodiazepenes, cocaine, methadone metabolite (EDDP) methamphetamine, 3,4-methylenedioxymethampetamine (MDMA), methadone, opiates, phencyclidine (PCP), oxycodone, tricyclic antidepressants, or THC). During the session, participants were asked about symptoms of withdrawal from cannabis, as assessed by the Marijuana Withdrawal Checklist (MWC; scored on a scale from 1 (lowest) to 4 (highest)) (Budney et al. 1999, Budney et al. 2003). The Pittsburgh Sleep Quality Index (Buysse et al. 1989) was also administered and the number of hours of sleep was recorded for the last week.

Participants then drove the simulator. Blood sample and oral fluid samples were collected at the time of the drive. Participants also completed cognitive and psychomotor testing, and subjective assessments (cognitive assessments and some subjective assessments to be published in a separate report). Self-report information was also collected during this session. Visual analog scales (VAS) were administered. The VAS measures were: ‘This feels like cannabis’ (CANN), ‘I like this drug effect’ (LIKE) and ‘I feel high’ (HIGH).

Before and after the drive, participants were asked about their willingness and ability to drive. For the ability to drive questions, participants were asked: (1) How well will you/did you drive during the simulation? (2) How much will/did the study drug affect your driving? Both of these questions were scored on a 6-point Likert-type scale labelled ‘not at all’, ‘slightly’, ‘somewhat well’, ‘moderately well’, ‘very well’, ‘extremely well’. Also in the ability questionnaire, participants were asked to rate confidence in their ability to drive and willingness to drive in specific environmental conditions. Adapted from the Adelaide Driving Self-Efficacy Scale (George et al. 2007), and using a 6-point scale labelled ‘not at all’, ‘slightly’, ‘somewhat’, ‘moderately’, ‘very’, ‘extremely’) participants were asked: In your current state, how CONFIDENT would you feel in your ability to drive: (a) in your own neighbourhood (within 10 min of home); (b) in an area that is unfamiliar to you; (c) in heavy traffic; (d) on a fast-moving road; (e) at night? For the willingness questions, participants were asked to answer on a 6-point scale (‘not at all’, ‘slightly’, ‘somewhat’, ‘moderately’, ‘very’, ‘extremely’): In your current state, how WILLING would you be to drive: (a) in your own neighbourhood (within 10 min of home); (b) in an area that is unfamiliar to you; (c) in heavy traffic; (d) on a fast-moving road; (e) at night?

### Cannabis

Participants in the cannabis group were asked to purchase cannabis from a legal retailer. They were permitted to choose their preferred cannabis. They were asked to record the time that they started and stopped smoking the cannabis and to arrive for the test session between 12 and 15 h after smoking. To facilitate an estimation of the amount smoked, participants were asked to smoke cannabis in the form of a new pre-roll. The ‘remnant’ of the pre-roll was brought to the lab and weighed. The amount smoked was estimated by subtracting the weight of the ‘remnant’ from the initial weight of the pre-roll (listed on the packaging). The weights of the filters were obtained from contacting the manufacturer, or purchasing the product separately and weighing the filter. For one product with a glass filter, an accurate weight was not possible so the weight of the filter was based on another glass filter for which a weight was obtained from the manufacturer. The packaging from the cannabis was also brought to the lab to record the details of the cannabis, including THC and CBD content, whether the product was infused with THC, and to verify that the cannabis was purchased legally. To estimate the amount of THC used in the joint, the net weight was multiplied by the percentage of THC as indicated on the package.

### Driving simulations

For details of the driving simulator (Virage VS500M – 2024 release), see our previous studies (Brands et al. 2019, Fares et al. 2022). Participants complete 4 driving scenarios: two measured speed and lateral control, one measured braking latency and the other assessed following distance. Collisions were also recorded by study personnel.

To assess speed and SDLP, participants drove two different scenarios, each lasting about 9 km (the time taken to complete this varied by participant). The participants drove on a two-lane rural highway, and were provided the opportunity to speed and race. To better simulate the cognitive demands of real-world driving conditions, one of these 10-min scenarios was conducted under dual task conditions, whereby the participant was asked to count backward by 3s from 900 in this single session (North and Hargreaves 1999).

To measure reaction time in terms of brake pedal latency, the scenario consisted of a 4-lane highway where participants were instructed to drive at 100 km/h, while remaining in the second lane to the right. When presented with a stop sign facing them, participants were to come to a complete stop as quickly as possible. When presented with a stop sign facing away from them, participants were to maintain their speed. During each trial a total of 10 stop signs appeared suddenly at the far right lane, 7 of which were facing them and 3 were turned away. The variable presented the mean of 7 ‘true’ stop signs.

In the following distance task, the participant drives through the scenario while following a lead vehicle. At the start, the lead vehicle is positioned roughly 35 m in front of the participant’s car. In the practice session, the car-following distance is shown on-screen as they drive to help the participant understand the distances. During the actual test session, this display is removed. After ignition, the lead vehicle accelerates to 60 km/hr within a few seconds and then varies its speed between 60 and 110 km/hr over the course of the drive. Participants are instructed to maintain the same following distance (35 m) throughout the scenario. Following distance measures are calculated using approximately 5 km of driving data, excluding the first and last 100 m, while speed utilizes about 9 km of driving data.

### Driving outcomes

- Standard deviation of lateral position (SDLP): Standard deviation of lateral position is the measure most consistently affected by cannabis (Alvarez et al. 2021, Brands et al. 2021). It measures the amount of ‘weaving’ (in centimetres), or lane deviation and gives an indication of the ability of a driver to maintain lateral control of the vehicle. In this study, SDLP was measured in meters.
- Speed: For the two scenarios measuring speed, participants were asked to maintain a speed of 80 km/hr. Cannabis generally produces decreases in speed (Alvarez et al. 2021, Brands et al. 2021), which are thought to be compensatory for perceived impairment (Ward and Dye 1999). In the present study, mean speed (MS) and maximal speed (MAX) were measured in km/hr. Standard deviation of speed (SDSP) was also measured, which represents the variability of speed during a drive. Larger numbers mean that the driver was not able to maintain a consistent speed.
- Brake latency/reaction time: This measured, in milliseconds, the time taken to hit the brake when a stop sign appeared on the right-hand side of the screen.
- Following distance: The participants were asked to follow a lead vehicle and to maintain a constant distance from that car. The speed of the lead car varied throughout the simulation. Measures collected were mean following distance (metres), minimum following distance (metres) and standard deviation of following distance, which gave an indication of the variability in maintaining the constant distance.
- Number of collisions: This is manually recorded by study personnel during the drives and consists of the number of times a vehicle collides with another car or any other object.

### Collection of blood and oral fluid

For quantification of THC, THC-COOH, 11-OH-THC and CBD, blood was collected directly into lavender top test tubes and then transferred to cryotubes for storage the same day to a -80 °C freezer until transfer to the partner lab (Dynacare) for analysis. Since participants were invited to use their preferred cannabis, we included CBD in the analysis because some of the participants would select products with CBD in them. Details of this analysis can be found in our previous report (Di Ciano et al. 2024). Oral fluid was collected in Quantisal vials and placed in a -80 °C freezer until transfer to the partner lab (Dynacare) for analysis. For all measures, the limit of quantification was 0.2 ng/mL. For participants with values below this limit, a value of 0.1 ng/mL was substituted (Beal 2001).

### Data analysis and sample size calculation

Statistical analysis was performed using statistical software R (v4.5) with packages “crosstable”, “emmeans”. Demographic data were analyzed descriptively with the package “crosstable”. Continuous data were analysed with t-tests or Wilcoxon rank sum test, depending on the distribution of the data. Count data were analysed with Fisher’s Exact Test or Chi-square, depending on the cell counts. Linear regression models were used with the driving outcome, subjective experience or driving ability/willingness as the dependent variables, and the grouping variable as the predictor, adjusting for sex, age and years of driving experience. The marginal means for each group, contrasts and effect sizes between the groups were estimated from the models. Correlation between the outcome measures and blood/oral fluid concentrations of THC/CBD or metabolites was analyzed and the Pearson correlation coefficients were reported. There were no missing data. P values were adjusted for multiple comparisons using the Benjamini-Hochberg false discovery rate (FDR) procedure with each family of tests controlled with a FDR set at 5%.

With the proposed sample size of 65 in each group, we have sufficient power (0.80) to detect a medium effect size (Cohen’s d = 0.50) between the cannabis user group and the control group on the outcome measures, or as small as 0.34 Pearson correlation between THC/metabolites and driving outcome in the cannabis user group.

## Results

**Fig. 1:**
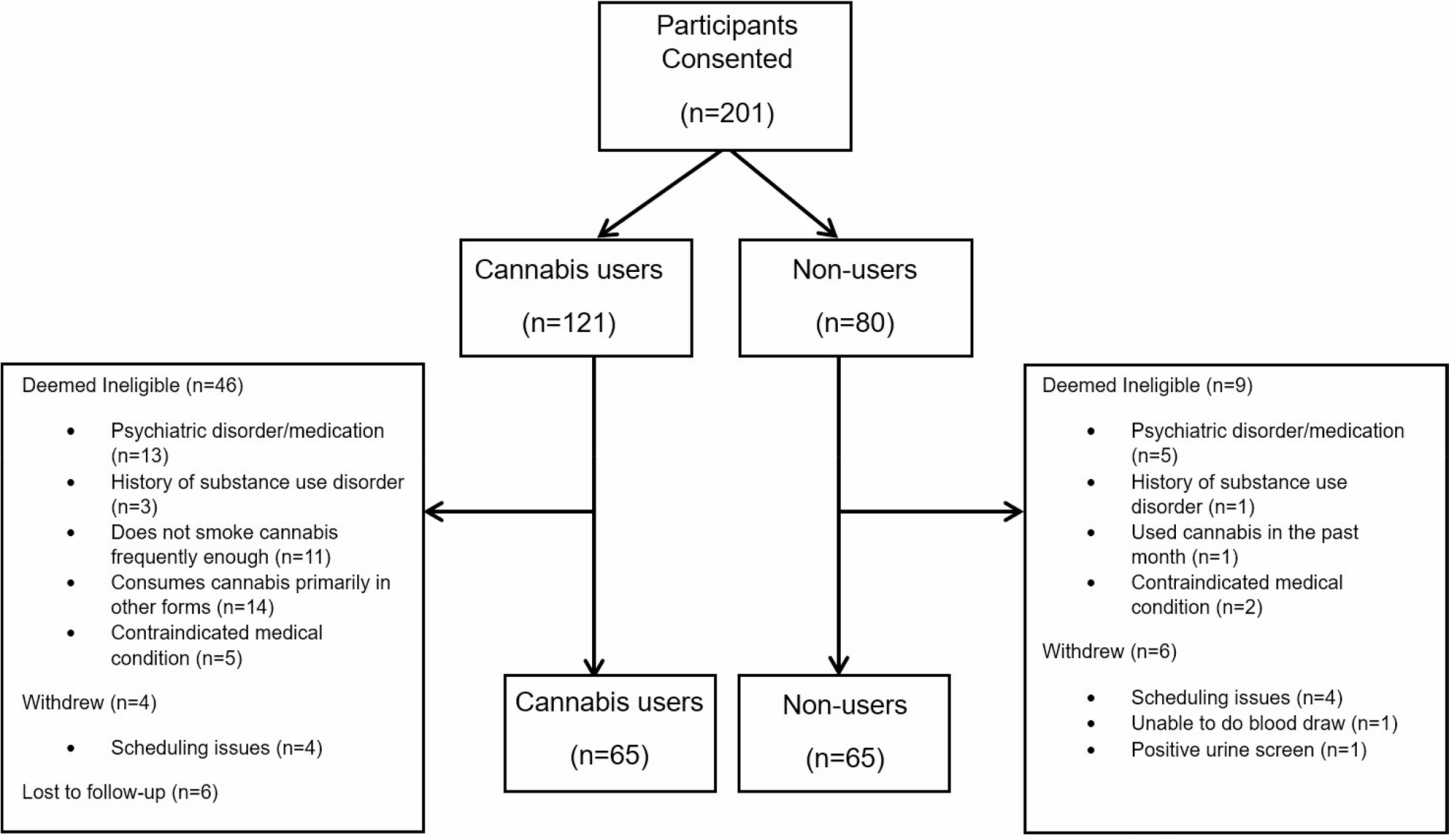
CONSORT diagram

In total, 201 participants were consented (cannabis group: *n* = 121). Of these, for the cannabis group, 46 were deemed ineligible, 4 withdrew and 6 were lost to follow-up. In the control group, 9 were deemed ineligible and 6 withdrew. In each group, 65 participants completed all study procedures. See Fig. 1 for a CONSORT diagram. Data was missing for 2 participants for amount of THC inhaled due to the fact that these participants did not bring the remnant of the ‘joint’ into the lab on the day of testing.

There were 33 males and 32 females in each of the groups (sex was based on birth-assigned sex). The cannabis group was 31 (7.2) years old (mean (standard deviation)), while the control group was on average 30 (6.6) years old. Their body mass index was matched (cannabis: 26 (5.0); control: 25 (4.2). The groups did not differ on the number of years they had been driving (cannabis: 12 (7.6); control: 10 (7.0), or kilometers driven in a year (cannabis: 10,513 (16,187); control: 11,421 (12,123) or in a week (cannabis: 208 (638); control: 105 (168). They also did not differ on the age when they received their full driver’s licence (cannabis: 19 (3.9); control: 20 (4.6)). The two groups reported the same amount of sleep in the past week (cannabis: 51 (5.4); control: 50 (7.1)) or the night before (cannabis: 7.3 (1.2); control: 7.0 (1.3)). Out of the 65 in each group, 8 in the control and 13 in the cannabis group drove after 12:00pm, with 9 of the 13 in the cannabis group being tested between 12:00–1:00pm. See Table 1.

**Table 1:** Demographics. Mean (SD); range. Continuous data were analysed with a two sample t-test or Wilcoxon rank sum test (depending on the distribution of the data). Count data were analysed with Fisher’s Exact Test or Chi-square (depending on the cell counts)

Variable	Cannabis	Control	*p* value
Age (years)	31 (7.2): 19–44	30 (6.6): 20-45	0.8395
Sex (Male/female)	33 / 32	33 / 32	1
Body Mass Index	26 (5.0); 18–41	25 (4.2); 17–43	0.359
Years Driving	12 (7.6); 1–26	10 (7.0); 0–27	0.1972
Km driven per year	10,513 (16,187); 0-100,000	11,421 (23,123); 0-140,000	0.3645
Km driven per week	208 (638); 0–5000	105 (168); 0–950	0.7979
Age of full licence (years)	19 (3.9); 15–35	20 (4.6); 15–45	0.0623
Hours of sleep in the last week	51 (5.4); 40–63	50 (7.1); 7.9–63	0.8208
Hours of sleep the night before	7.3 (1.2); 3–10	7.0 (1.3); 3–10	0.3018
Race			
· Asian	8 (12.3%)	40 (62%)	< 0.0001
· Black	6 (9.2%)	5 (7.7%)	
· Indigenous	3 (4.6%)	1 (1.5%)	
· Latin American	4 (6.2%)	5 (7.7%)	
· Middle Eastern	2 (3.1%)	1 (1.5%)	
· White	36 (55%)	13 (20%)	
· Mixed	4 (6.2%)	0 (0%)	
· Other	2 (3.1%)	0 (0%)	
Highest Education			
· Elementary/High School	13 (20%)	4 (6.2%)	< 0.0001
· College/technical	38 (59%)	10 (15%)	
· University (Undergraduate)	0 (0.0%)	27 (42%)	
· University (Advanced)	14 (22%)	24 (37%)	
THC % smoked	30 (6.7): 9.4–45	N/A	N/A
CBD % smoked	0.6 (2.5): 0–14	N/A	N/A
THC inhaled	159 (90); 7.2–385		
Frequency of cannabis use		N/A	N/A
· 3-4x per week	8 (12%)		
· 5-6x per week	13 (20%)		
· 1x per day	24 (37%)		
· More than once a day	20 (31%)		
Number of participants smoking infused cannabis		N/A	N/A
· No	48 (74%)		
· Yes	17 (26%)		
Number of years using cannabis	12 (7.1): 1–26	N/A	N/A
Potency of cannabis normally used		N/A	N/A
· 0–4%	1 (1.5%)		
· 5–9%	0 (0.0%)		
· 10–14%	2 (3.1%)		
· 15–19%	5 (7.7%)		
· 20–24%	25 (39%)		
· 25–30%	26 (40%)		
· >30%	6 (9.2%)		
Time of last cannabis use		N/A	N/A
· 6pm-7pm	1 (1.5%)		
· 7:01-8pm	5 (7.7%)		
· 8:01-9pm	7 (11%)		
· 9:01-10pm	22 (34%)		
· 10:01-11pm	15 (23%)		
· 11:01-12pm	10 (15%)		
· 12:01am-1am	5 (7.7%)		
Time since last cannabis use (hours)	13 (0.9); 12.2–15.4	N/A	N/A
Marijuana withdrawal checklist total	1.2 (0.2); 1–2	N/A	N/A
Marijuana withdrawal checklist withdrawal discomfort	1.2 (0.2); 1-1.9	N/A	N/A
Blood THC	2.8 (4): 0.1 − 18	0.1(0):0.1	N/A
Blood THC-COOH	29 (45): 0.1–225	0.1(0):0.1	
Blood THC-11-OH	6.9 (15): 0.1–82	0.1 (0.03): 0.1-0.3	
Blood CBD	0.3 (1): 0.1–7.6	0.1(0):0.1	
Oral fluid THC	31 (75): 0.1–399	0.1(0):0.1	
Oral fluid CBD	0.3 (0.9): 0.1–5.6	0.1(0):0.1	

The two groups differed only in race and highest education attained. In the cannabis group, 36 (55%) of the participants were white, with 2 to 8 participants in each of the categories of Asian, Black, Indigenous, Latin American, Middle Eastern, mixed race or other. In the control group, 40 participants (62%) were Asian and 13 (20%) were white, with 0 to 5 in each of the other categories (*p* < 0.001). The control group had completed more formal education than the cannabis group; 27 (42%) and 24 (37%) had an Undergraduate or Graduate degree, respectively. By comparison, 38 (59%) of the cannabis group reported college or technical school (*p* < 0.001). See Table 1.

The cannabis group smoked cannabis, with on average, 30 (6.7)% THC and 0.6 (2.5)% CBD. The mean amount of THC inhaled was approximately 159 (90) mg. Of the 65, 17 (26%) chose infused products. Eight (12%) endorsed cannabis use 3–4 times per week, while 13 (20%) smoked cannabis 5–6 times per week. Twenty-four (37%) and 20 (31%) reported daily or more than daily use of cannabis, respectively. On average, they had been using cannabis for 12 (7.1) years. Most regularly used high potency cannabis, with 25 (39%) and 26 (40%) reporting they usually smoke cannabis of 20–24% or 25–30%, respectively; only 6 (9.2%) regularly used cannabis with THC above 30%. The participants smoked cannabis on average 13 (0.9) hours prior to driving the simulator and most (*n* = 22) smoked between 9:01–10:00pm, with 15 smoking between 10:01–11:00pm and 10 smoking between 11:01pm-12:00am. They reported minimal withdrawal symptoms during the test session, as measured by the Marijuana Withdrawal Checklist, with a rating scale from 1 to 4 (4 being the most withdrawal). The total score was 1.2 (0.2). The withdrawal discomfort score was 1.2 (0.2). See Table 1.

### Driving

After adjusting for age, sex, and years of driving experience, comparison of the estimated marginal mean for SDLP for the cannabis group to the control group revealed a small yet significant effect for both the standard task and dual task conditions (standard: t(125)=-2.185, *p* = 0.031, effect size Cohen’s d = 0.389; dual task: t(125)=-2.015, *p* = 0.046, effect size Cohen’s d=-0.359). These comparisons were not significant after correction for multiple comparisons (*p* = 0,277 for each test). The comparison of the two groups demonstrated no statistically significant difference for any other measure of driving. The number of collisions was too few to permit analysis. See Table 2. Driving was not impacted by smoking high potency infused cannabis. See Table 3. 

**Table 2:**
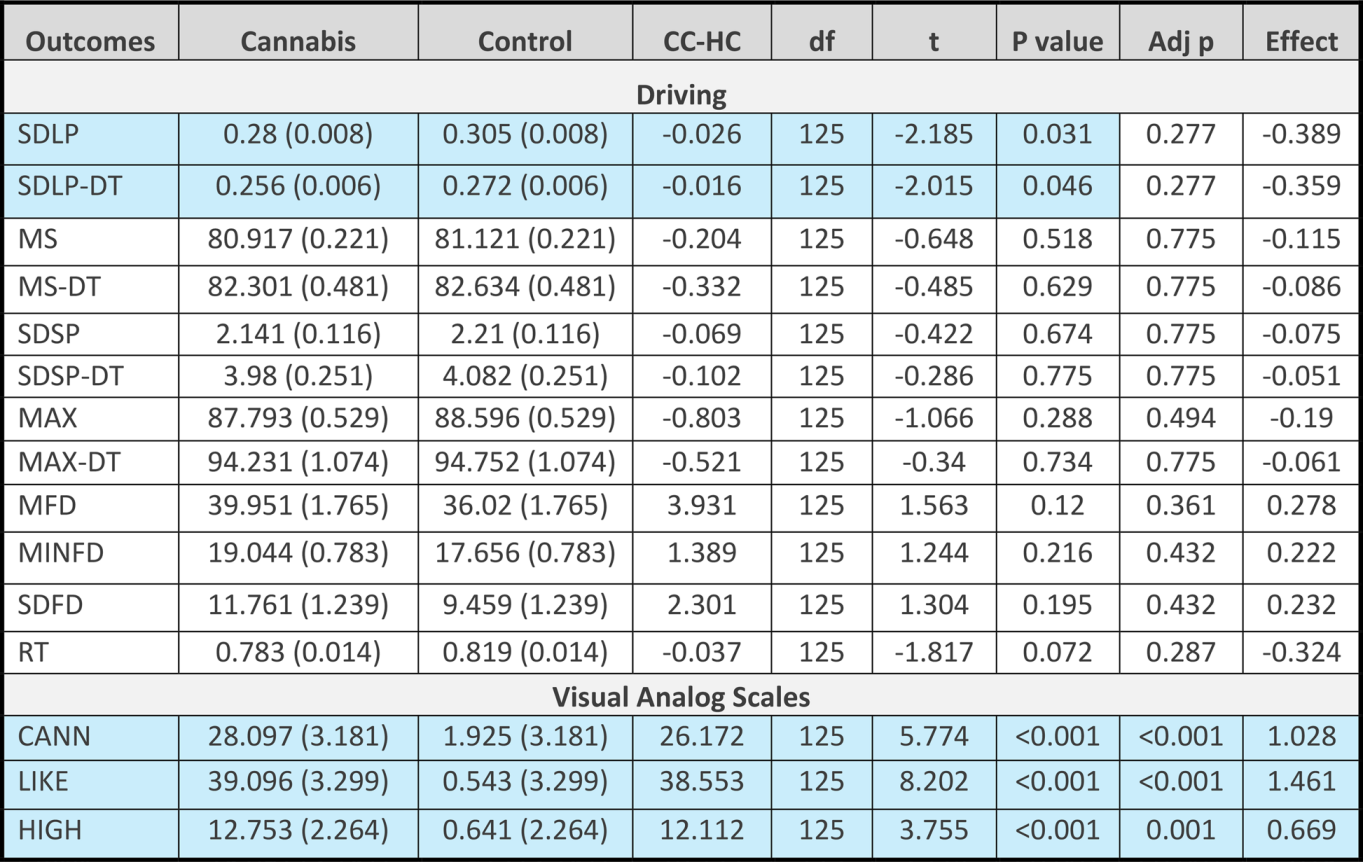
Driving and visual analog scale outcomes (adjusted) for the cannabis (CC) and control (HC) groups

*CC-HC* Contrast; Effect: Cohen’s d, *SDLP(-DT)* Standard deviation of lateral position (dual task), *MS(-DT)* Mean speed (dual task), *SDSP(-DT)* Standard deviation of speed (dual task), *MAX(-DT)* Maximal speed (dual task), *MFD* Mean following distance, *MNFD* minimum following distance, *SDFD* Standard deviation of following distance, *RT* Reaction time/brake latency, *CANN* ‘This feels like cannabis’, *LIKE* ‘I like this drug effect’, *HIGH* ‘I feel high’. Adj p = *p* value after correction for multiple comparisons

Blue cells represent differences between the CC and HC groups (*p*<0.05)

**Table 3:** Effect of use of infused cannabis on driving outcomes (adjusted). No: Did not use infused cannabis’ Yes: Did use infused cannabis. No-Yes: contrast; Effect: Cohen’s d

Outcomes	No	Yes	No-Yes	df	t	*P* value	Adj *p*	Effect
SDLP	0.276 (0.009)	0.285 (0.016)	-0.009	60	-0.472	0.639	0.945	-0.136
SDLP-DT	0.253 (0.006)	0.257 (0.011)	-0.003	60	-0.271	0.788	0.945	-0.078
MS	81.053 (0.227)	80.567 (0.385)	0.486	60	1.079	0.285	0.945	0.311
MS-DT	82.335 (0.479)	82.315 (0.813)	0.021	50	0.022	0.983	0.983	0.006
SDSP	2.111 (0.125)	2.244 (0.212)	-0.134	60	-0.539	0.592	0.945	-0.156
SDSP-DT	3.838 (0.298)	4.305 (0.506)	-0.467	60	-0.789	0.433	0.945	-0.228
MAX	87.975 (0.512)	87.261 (0.870)	0.714	60	0.702	0.486	0.945	0.202
MAX-DT	94.394 (1.279)	93.603 (2.173)	0.791	60	0.311	0.757	0.945	0.09
MFD	40.76 (2.36)	37.09 (4.01)	3.67	60	0.782	0.437	0.945	0.226
MINFD	19.492 (0.904)	17.799 (1.537)	1.693	60	0.942	0.35	0.945	0.272
SDFD	11.689 (1.933)	11.847 (3.284)	-0.158	60	-0.041	0.967	0.983	-0.012
RT	0.779 (0.015)	0.789 (0.025)	-0.01	60	-0.332	0.741	0.945	-0.096

*SDLP(-DT)* Standard deviation of lateral position (dual task), *MS(-DT)* Mean speed (dual task), *SDSP(-DT)* Standard deviation of speed (dual task), *MAX(-DT)* Maximal speed (dual task), *MFD* Mean following distance, *MNFD* Minimum following distance, *SDFD* Standard deviation of following distance, *RT* Reaction time/brake latency. Adj p = *p* value after correction for multiple comparisons

### Subjective experience and driving ability/willingness

After adjusting for age, sex, and years of driving experience, comparison of the estimated marginal mean for the cannabis group to the control group revealed significantly higher ratings in the cannabis group for all VAS measures, with large effect sizes for ‘this feels like cannabis’ and ‘I like this drug effect’, and a medium effect for ‘I feel high’. The comparisons remained significant after correction for multiple comparisons (this feels like cannabis: t(125) = 5.774, p < 0.001; Cohen’s d = 1.028; adjusted p < 0.001; I like this drug effect: t(125) = 8.202, p < 0.001, Cohen’s d = 1.462; adjusted p < 0.001; I feel high: t(125)-3.755, p < 0.001, Cohen’s d = 0.669; adjusted p = 0.001). See Table 2.

When asked about willingness and ability to drive, only one comparison remained significant after correction for multiple comparisons. When asked “how much will/did the study drug affect your driving”, the cannabis group rated the effect as higher after the drive but difference before the drive was not significant after correction for multiple comparisons. Both were medium-sized effects (before: (t(125) = 2.879, *p* < 0.005, Cohen’s d = 0.513, adjusted *p* = 0.513; after: t(125) = 3.822, *p* < 0.001; Cohen’s d = 0.681, adjusted *p* = 0.005). For “how well will you/did you drive during the simulation”, participants in the cannabis group rated their ability as lower after the drive, but the effect was small and was not significant after correction for multiple comparisons (t(125)=-1.999, *p* = 0.048, Cohen’s d: 0.356, adjusted *p* = 0.213). When asked about their willingness to drive, the cannabis group rated their willingness to drive in their own neighbourhood, in heavy traffic and at night, higher than the control group before driving (with small-sized effects), but these differences were not significant after corrections for multiple comparisons (own neighbourhood: t(125) = 1.986, *p* = 0.049, Cohen’s d = 0.354, adjusted *p* = 0.213; in heavy traffic: t(125) = 2.323, *p* = 0.022, Cohen’s d = 0.414, adjusted *p* = 0.189; at night: t(125) = 2.134, *p* = 0.035, Cohen’s d = 0.38, adjusted *p* = 0.213). After driving there were no differences between groups in reported willingness to drive. See Table 4 for the measures before driving and Table 5 for measures after driving.

**Table 4:**
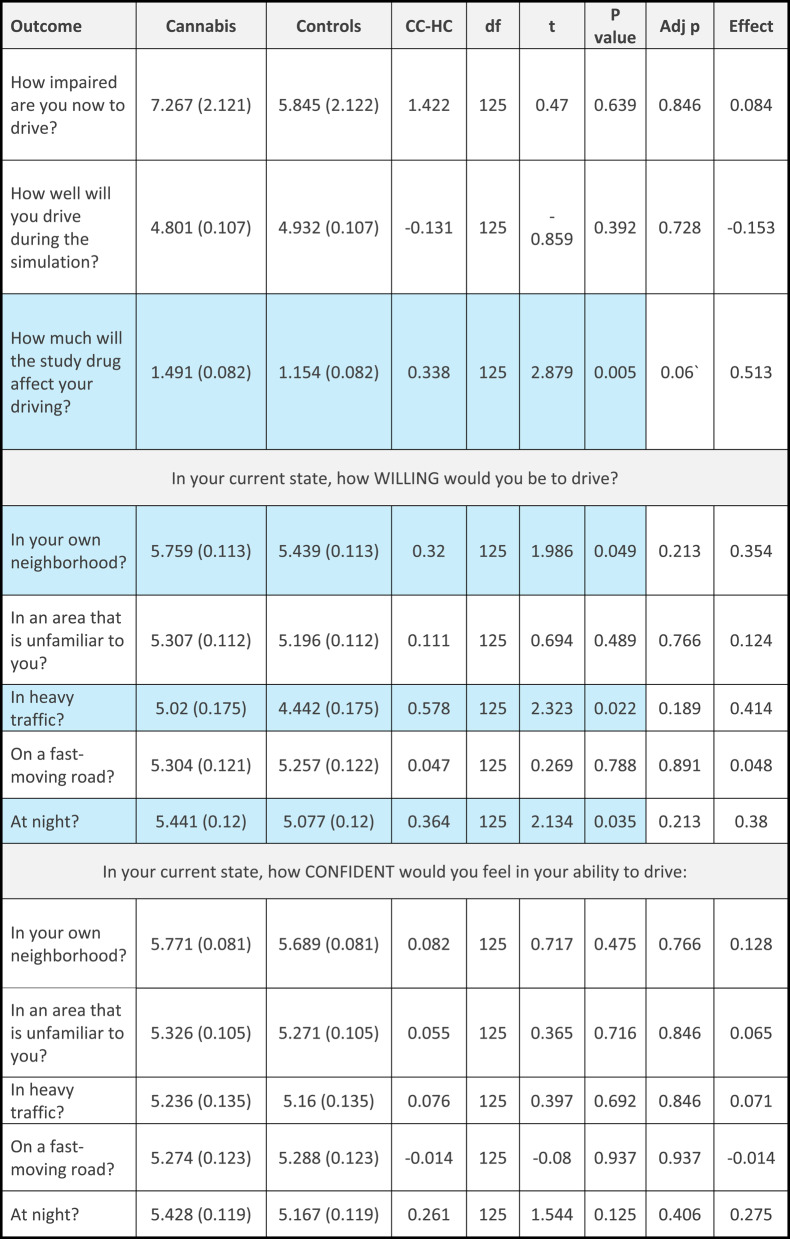
Willingness and ability to drive before the simulated drive (adjusted) in the cannabis (CC) and control (HC) group

*CC-HC* Contrast; Effect: Cohen’s d. adj p = *p* value after correction for multiple comparisons

Blue cells represent differences between the groups (*p*<0.05)

**Table 5:**
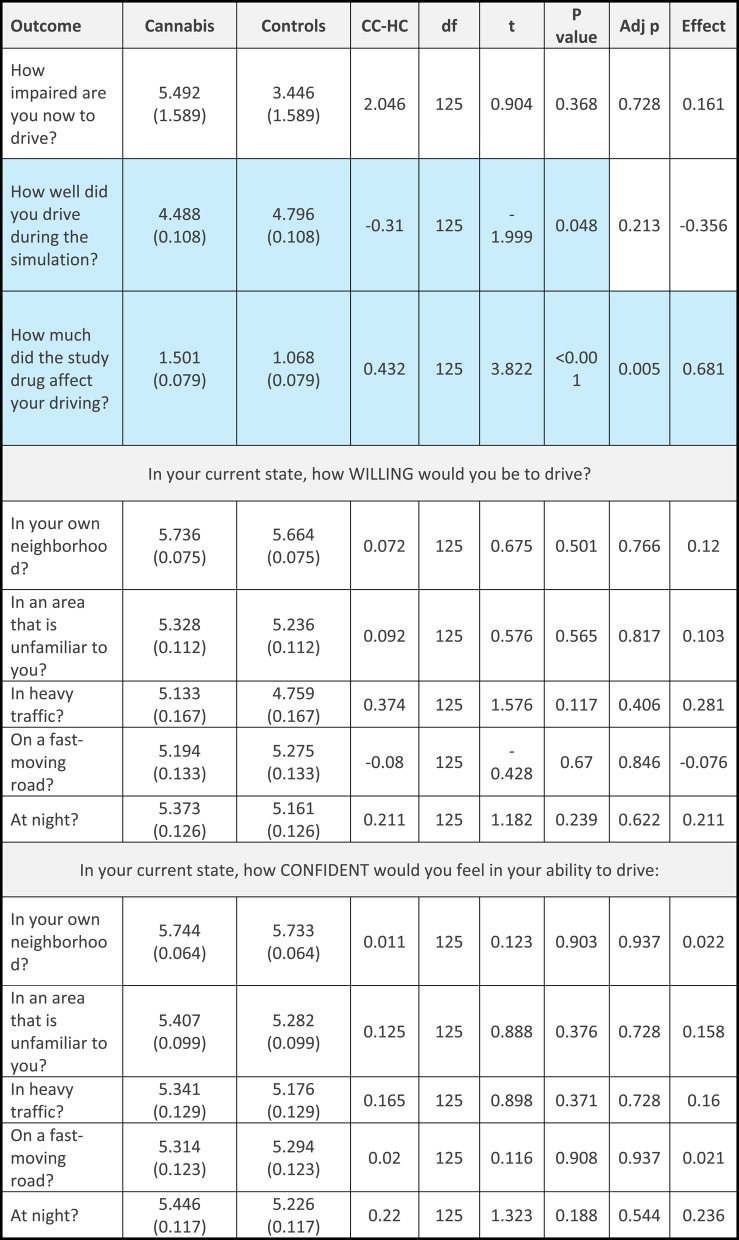
Willingness and ability to drive after the simulated drive (adjusted) in the cannabis (CC) and control (HC) group

*CC-HC* Contrast; Effect: Cohen’s d. adj p = *p* value after correction for multiple comparisons

Blue cells represent differences between the groups (*p*<0.05)

### Association of THC and metabolites to driving

In the cannabis group, blood THC the morning after smoking cannabis was at a mean concentration of 2.8 (4) ng/mL. The median (Interquartile Range) was 1.2 (0.5; 2.7). The metabolites THC-COOH and THC-11-OH were at a mean concentration of 29 (45) ng/mL and 6.9 (16) ng/mL, respectively. Concentrations of CBD in blood were low, at 0.3 (1) ng/mL. THC in oral fluid was on average 31 (75) ng/mL, and oral fluid CBD was 0.3 (0.9) ng/mL. Metabolites of THC in OF were below the limit of quantification. The control group was below the limit of quantification for all measures. See Table 1.

Pearson’s correlation between driving variables and THC or CBD in the ‘joint’ smoked, the amount of THC inhaled, or between THC in blood and oral fluid and metabolites in blood revealed a few small, yet significant effects. The most consistent effect was with the active metabolite 11-OH-THC, where trends to positive correlations were found between blood concentrations of 11-OH-THC and SDLP after correction for multiple comparisons (*r* = 0.294, *p* = 0.018, adjusted *p* = 0.071), mean following distance after correction for multiple comparisons (0.284, *p* = 0.022, adjusted *p* = 0.071) and standard deviation of following distance after correction for multiple comparisons (0.290, *p* = 0.019, adjusted *p* = 0.071). A positive correlation of reaction time to oral fluid THC was also seen that was not significant after correction for multiple comparisons (*r* = 0.286, *p* = 0.021, adjusted *p* = 0.136). In addition, there was a positive correlation between percent CBD in the cannabis and SDLP under dual task conditions that was not significant after correction for multiple comparisons (*r* = 0.261, *p* = 0.036, adjusted *p* = 0.432). No other relationships were significant. See Table 6. 

**Table 6:**
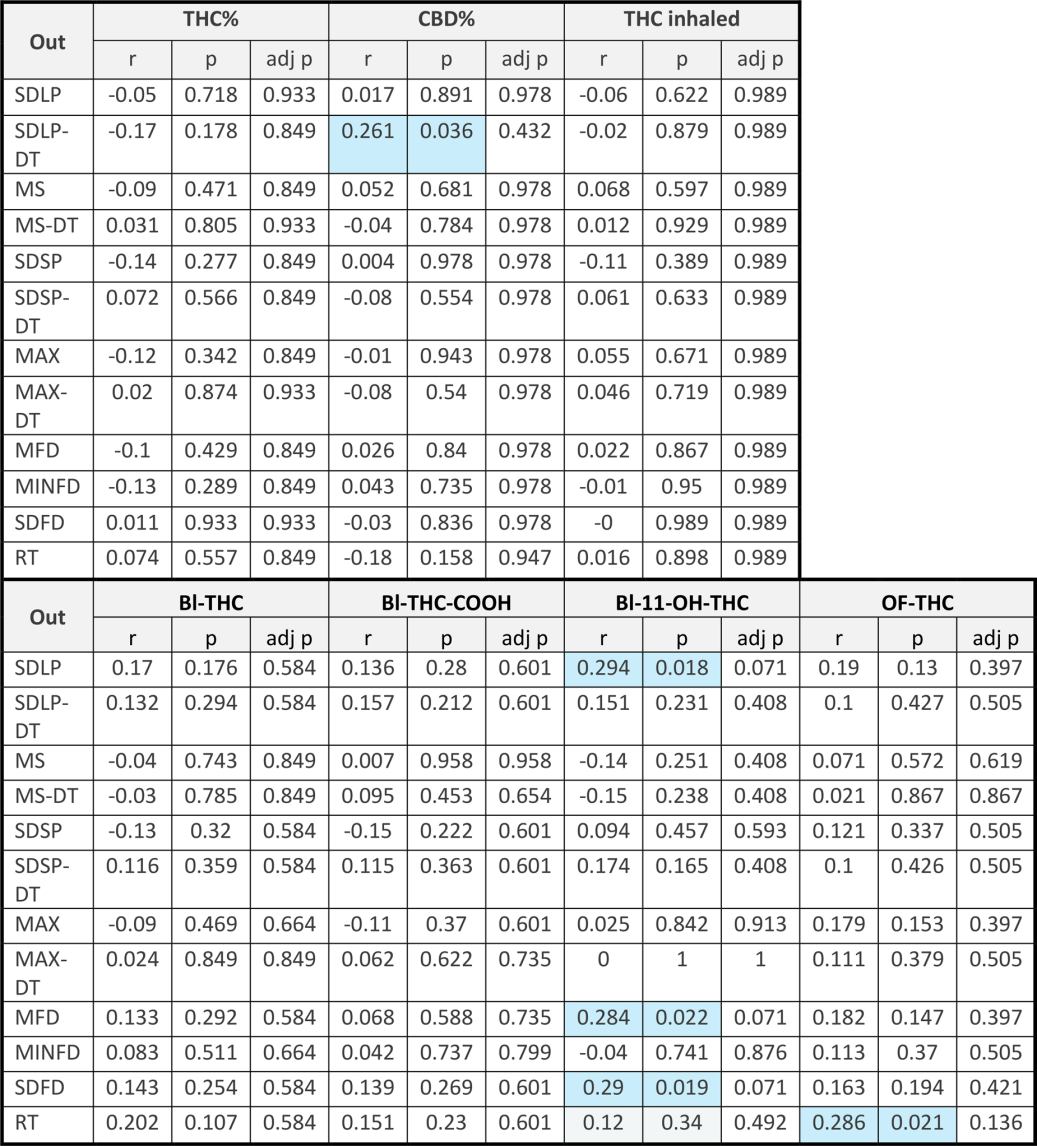
Correlations between potency of THC (THC%) or CBD (CBD%) and driving variables and between blood (Bl) or oral fluid (OF) THC or metabolites and driving variables

*SDLP(-DT)* Standard deviation of lateral position (dual task), *MS(-DT)* Mean speed (dual task), *SDSP(-DT)* Standard deviation of speed (dual task), *MAX(-DT)* Maximal speed (dual task), *MFD* Mean following distance, *MNFD* minimum following distance, *SDFD* Standard deviation of following distance, *RT* Reaction time/brake latency, *Out* Outcome, *Bl* Blood, *OF* Oral fluid. adj p = *p* value after correction for multiple comparisons

Significant correlations (*p*<0.05) are marked by blue cells

## Discussion

The purpose of the present study was to determine whether there were differences in driving 12–15 h after using cannabis at night in people who use smoked cannabis frequently, relative to a control group that did not use cannabis. It was found that SDLP in both the standard and dual task conditions was not significantly different in the cannabis users compared to the non-cannabis users after correction for multiple comparisons. No other driving measures of speed, following distance or reaction time were significantly different between groups. A tendency for cannabis users to be more willing to drive before the drive than controls was seen, but this was not significant after correction for multiple comparisons. Cannabis users reported significantly higher ratings on the visual analog scales than controls, even though they had consumed cannabis 12–15 h prior to testing. The percentage of THC or CBD reported in the smoked cannabis was not correlated with any measure of driving, and use of high-potency infused cannabis also was not associated with these measures. As well, there were no correlations between any driving measure and blood THC or blood THC-COOH. Some relationships between oral fluid THC and reaction time, and between blood 11-OH-THC and SDLP, mean following distance and standard deviation of following distance, were found that were not significant after correction for multiple comparisons.

The finding that driving was not different between groups is not consistent with previous reports which found that driving was negatively impacted in frequent users (Doroudgar et al. 2018, Dahlgren et al. 2020) or occasional users (Hartley et al. 2019). Some studies found no evidence of residual impairment at 24 to 48 h after last use (Brands et al. 2019, Mastropietro et al. 2025, Ronen et al. 2010, Ronen et al. 2008), which is consistent with the present findings. One reason for the discrepancy in findings could be due to the fact that in one study (Dahlgren et al. 2020), impairment was limited to the participants with early onset of cannabis use (before age 16). In the present sample, participants were on average 30 years old and the mean number of years of using cannabis was 11.64, thus it may be possible that the current sample did not capture people who started using cannabis at an early age. In the other study (Doroudgar et al. 2018), driving was impaired on measures that were not captured in the present study, including modulus and coherence. In another study, changes in driving were apparent only in occasional users (Hartley et al. 2019). Future studies with a more comprehensive battery of tests may capture residual changes in driving. This highlights the importance of testing the effects of cannabis on diverse populations using diverse driving scenarios and simulators. Subtle residual effects of cannabis may be apparent in subgroups of users.

There is no doubt that cannabis can affect driving in the hours after last use of cannabis. However, the exact duration of these effects remains to be determined. Most studies of the acute effects of cannabis have investigated the effects of cannabis within a few hours of use (Brands et al. 2021, Arkell et al. 2020). Some studies with longer durations (e.g. 24 h) have reported no effects (Brands et al. 2019). The present study suggests that frequent users of cannabis may not be impaired at 12–15 h after using cannabis. It is known that the time course of effects of other routes of administration may differ from those of inhaled routes (Behzad et al. 2025). Notably, the effects of edibles on blood and oral fluid THC can last longer (Spindle et al. 2021), suggesting that the effects of oral routes on driving may persist longer. However, at this time there are only two published reports of the effects of edibles on driving (Zhao et al. 2024, Won et al. 2025), and effects were measured only up to 6 h after ingestion. Future studies will need to investigate the time course of effects of various methods of cannabis use.

The users in the present study were frequent users of cannabis. Thus, there may have been some tolerance to the effects of cannabis, which may explain the lack of impairment of driving in the cannabis group relative to the control group. However, other papers of residual effects looked at performance of frequent users (Doroudgar et al. 2018, Dahlgren et al. 2020) and found decrements in residual performance. Indeed, the evidence for tolerance in the acute effects of cannabis on driving is mixed. In one study, driving impairments were worse in regular cannabis users compared to non-regular users after smoking cannabis (Downey et al. 2013). In another study, ‘weaving’ was more evident in non-regular users, as compared to regular users after oral synthetic cannabis (dronabinol) (Bosker et al. 2012). In a more recent study, occasional users demonstrated more lane departures while distracted, with few differences from habitual users in any other measures while not distracted (Miller et al. 2022). Future studies will need to investigate the residual effects of cannabis in occasional and infrequent users, who may need to exercise caution when operating a motor vehicle the morning after last use.

In the present study, the cannabis group apparently demonstrated residual intoxication in the visual analog scale the morning after last use of cannabis. It may seem alarming that participants who report intoxication may also not be impaired at driving and may report that the drug affected their ability after the drive. However, the increase in visual analog scale score, although different from the control group, only reached maximal levels of about 30 out of 100. This is similar to the magnitude of placebo effects that we have observed in past studies (Matheson et al. 2019). Thus, it cannot be discounted that demand characteristics may have factored into the present ratings on the visual analog scale. An additional consideration is the fact that there was no baseline control, meaning that differences between groups could be due to pre-existing differences between the groups. Inclusion of a baseline would have been difficult because the cannabis group used cannabis at least 4 times a week (with many reporting daily use) and it would have been difficult for this group to abstain for a number of days. In any event, the groups were fairly well matched, with no differences in age, sex, years of driving experience, the amount of driving per week/year and the number of hours slept the night before/past week. Differences in race and education pose interesting questions for future investigations into the characteristics of cannabis-induced intoxication.

In the present study, there were no correlations between blood or oral fluid THC and any measure of driving. Deterrence of driving after the use of cannabis rests in part on the detection of THC in blood and oral fluid. It should be considered that some recent studies have cast doubt on a simple relationship between THC and driving ability (Fitzgerald et al. 2023, Marcotte et al. 2022). Indeed, in our systematic review (Behzad et al. 2025), we found that all but two studies (Hartman et al. 2016, Hartman et al. 2015) found no linear correlation between blood THC and measures of driving performance. In a past study of residual effects of cannabis, measures of driving performance were negatively impacted in participants with blood THC above 2 or 5 ng/mL (Doroudgar et al. 2018). However, as discussed above, that study used measures of driving performance not captured in the present study. In the present study, mean blood concentrations of THC were above 2 ng/mL, the legal limit in some jurisdictions. Despite this, there was no evidence for a decrement in driving performance after use of cannabis. Thus, drivers may exceed legal limits for cannabis despite a lack of impairment. It should be considered that some participants had concentrations of THC below the limit of quantification and the median THC was 1.2 ng/mL, which is consistent with a lack of effect on driving. However, a number of participants were above the legal limit and no changes in driving were apparent. Future studies should continue investigating the utility of the standard 2 or 5 ng/mL cut-offs of blood THC for defining impaired driving.

This study is not without its limitations. Foremost, the observational design of this study precluded verification that the cannabis group smoked their cannabis at the time indicated. Further, it is difficult to verify exactly how much cannabis they smoked. Studies with a 12–15 h window before testing in the morning are difficult to conduct without providing overnight accommodation for the participants, and necessitating a much more complicated design. Nevertheless, use of observational designs is increasing and they are gaining more acceptance. Observational designs provide real-world applicability of data and provide better external validity than controlled laboratory studies. Together, with traditional clinical research approaches, naturalistic designs can help to provide a rich understanding of the features of cannabis use. 

## Conclusions

The present study found that people who use cannabis frequently and smoke it the night before driving did not demonstrate a decrement in driving performance relative to a control group. Residual mean concentrations of blood THC were above the legal limit of 2 ng/mL, suggesting that users may test positive for cannabis in the absence of any observed decrements in driving performance. Future studies will need to further investigate the relationship between blood and oral fluid THC and driving. Future large-scale studies are also needed to determine whether people who use cannabis less frequently are impaired by cannabis when they smoke cannabis the night before. Users should also be aware that different routes of administration (e.g. oral) may have more prolonged effects on driving and blood and oral fluid THC.

## Data Availability

The datasets used and/or analysed during this study are available from the corresponding author on reasonable request.

## Abbreviations

CAMH: Centre for Addiction and Mental Health
CANN: This feels like cannabis
CBD: Cannabidiol
HIGH: I feel high
LIKE: I like this drug effect
MAX: Maximal Speed
MS: Mean Speed
MWC: Marijuana Withdrawal Checklist
OF: Oral Fluid
REDCap: Research Electronic Data Capture
SDLP: Standard Deviation of Lateral Position
SDSP: Standard Deviation of Speed
THC: Delta-9-Tetrahydrocannabinol
THC- COOH: 11-Nor-9-carboxy-THC
VAS: Visual Analog Scale
11-OH-THC: 11-Hydroxy-THC

## References

[CR1] Hammond D et al. International Cannabis Policy Study - Canada 2022 Summary. 2023.

[CR2] Canada H. Canadian Cannabis Survey. 2024 March 25, 2024]; Available from: https://www.canada.ca/en/health-canada/services/drugs-medication/cannabis/research-data/canadian-cannabis-survey-2024-summary.html.

[CR3] Kucera A, Hammond D. Cannabis and driving: A repeat cross-sectional analysis of driving after cannabis use pre- vs. post-legalization of recreational cannabis in Canada. Addict Behav. 2025;170:108419. 40618444 10.1016/j.addbeh.2025.108419

[CR4] Hostiuc S, et al. The Association of Unfavorable Traffic Events and Cannabis Usage: A Meta-Analysis. Front Pharmacol. 2018;9:99. 29487531 10.3389/fphar.2018.00099 PMC5816577

[CR5] Brubacher JR, et al. Cannabis Legalization and Detection of Tetrahydrocannabinol in Injured Drivers. N Engl J Med. 2022;386(2):148–56. 35020985 10.1056/NEJMsa2109371

[CR6] Jin A, et al. Cannabis consumption and motor vehicle collision: A systematic review and meta-analysis of observational studies. Int J Drug Policy. 2025;142:104832. 40367728 10.1016/j.drugpo.2025.104832

[CR7] Preuss UW, et al. Cannabis Use and Car Crashes: A Review. Front Psychiatry. 2021;12:643315. 34122176 10.3389/fpsyt.2021.643315 PMC8195290

[CR8] Ward NJ, Dye L. Cannabis and driving: a literature review and commentary. UK DETR Road Safety Research Report; 1999. p. 12.

[CR9] Alvarez L, et al. Young and under the influence: A systematic literature review of the impact of cannabis on the driving performance of youth. Accid Anal Prev. 2021;151:105961. 33421731 10.1016/j.aap.2020.105961

[CR10] Brands B, Ciano PD, Mann RE. Cannabis, Impaired Driving, and Road Safety: An Overview of Key Questions and Issues. Front Psychiatry. 2021;12:641549. 34489746 10.3389/fpsyt.2021.641549 PMC8416748

[CR11] Arkell TR, et al. Effect of Cannabidiol and Delta9-Tetrahydrocannabinol on Driving Performance: A Randomized Clinical Trial. JAMA. 2020;324(21):2177–86. 33258890 10.1001/jama.2020.21218 PMC7709000

[CR12] McCartney D, Suraev A, McGregor IS. The Next Day Effects of Cannabis Use: A Systematic Review. Cannabis Cannabinoid Res. 2023;8(1):92–114. 36475998 10.1089/can.2022.0185 PMC9940812

[CR13] Brands B, et al. Acute and residual effects of smoked cannabis: Impact on driving speed and lateral control, heart rate, and self-reported drug effects. Drug Alcohol Depend. 2019;205:107641. 31678833 10.1016/j.drugalcdep.2019.107641

[CR14] Doroudgar S, et al. Effects of chronic marijuana use on driving performance. Traffic Inj Prev. 2018;19(7):680–6. 30411981 10.1080/15389588.2018.1501800

[CR15] Dahlgren MK, et al. Recreational cannabis use impairs driving performance in the absence of acute intoxication. Drug Alcohol Depend. 2020;208:107771. 31952821 10.1016/j.drugalcdep.2019.107771 PMC9036916

[CR16] Hartley S, et al. Effect of Smoked Cannabis on Vigilance and Accident Risk Using Simulated Driving in Occasional and Chronic Users and the Pharmacokinetic-Pharmacodynamic Relationship. Clin Chem. 2019;65(5):684–93. 30872375 10.1373/clinchem.2018.299727

[CR17] Mastropietro KF, et al. Short-term residual effects of smoked cannabis on simulated driving performance. Psychopharmacology (Berl); 2025. 10.1007/s00213-025-06880-1 PMC12478537 40913146

[CR18] Ronen A, et al. The effect of alcohol, THC and their combination on perceived effects, willingness to drive and performance of driving and non-driving tasks. Accid Anal Prev. 2010;42(6):1855–65. 20728636 10.1016/j.aap.2010.05.006

[CR19] Ronen A, et al. Effects of THC on driving performance, physiological state and subjective feelings relative to alcohol. Accid Anal Prev. 2008;40(3):926–34. 18460360 10.1016/j.aap.2007.10.011

[CR20] Gjerde H, Strand MC. Legal limits for driving under the influence of illicit drugs: Large variations between jurisdictions. Forensic Sci Int: Rep. 2023;8:100336.

[CR21] Robbe H. Marijuana’s impairing effects on driving are moderate when taken alone but severe when combined with alcohol. Human Psychopharmacol. 1998;13:S70–8.

[CR22] Ramaekers JG, Robbe HW. O’Hanlon, *Marijuana, alcohol and actual driving performance*. Hum Psychopharmacol. 2000;15(7):551–8. 12404625 10.1002/1099-1077(200010)15:7<551::AID-HUP236>3.0.CO;2-P

[CR23] Lenne MG, et al. The effects of cannabis and alcohol on simulated arterial driving: Influences of driving experience and task demand. Accid Anal Prev. 2010;42(3):859–66. 20380913 10.1016/j.aap.2009.04.021

[CR24] Downey LA, et al. The effects of cannabis and alcohol on simulated driving: Influences of dose and experience. Accid Anal Prev. 2013;50:879–86. 22871272 10.1016/j.aap.2012.07.016

[CR25] Fitzgerald RL, et al. Driving Under the Influence of Cannabis: Impact of Combining Toxicology Testing with Field Sobriety Tests. Clin Chem. 2023;69(7):724–33. 37228223 10.1093/clinchem/hvad054 PMC10320013

[CR26] Marcotte TD, et al. Driving Performance and Cannabis Users’ Perception of Safety: A Randomized Clinical Trial. JAMA Psychiatry; 2022. 10.1001/jamapsychiatry.2021.4037 PMC8792796 35080588

[CR27] Behzad D et al. Association of driving with blood delta-9-tetrahydrocannabinol: a systematic review. Int J Neuropsychopharmacol. 2025;28(4):pyaf021. 10.1093/ijnp/pyaf021 PMC12032524 40172477

[CR28] Di Ciano P, et al. The Utility of THC Cutoff Levels in Blood and Saliva for Detection of Impaired Driving. Cannabis Cannabinoid Res. 2023;8(3):408–13. 36730769 10.1089/can.2022.0187

[CR29] Cuschieri S. The STROBE guidelines. Saudi J Anaesth. 2019;13(Suppl 1):S31–4. 30930717 10.4103/sja.SJA_543_18 PMC6398292

[CR30] Harris PA, et al. Research electronic data capture (REDCap) - A metadata-driven methodology and workflow process for providing thranslational researchinformatics support. J Biomed Inf. 2009;42(2):377–81. 10.1016/j.jbi.2008.08.010 PMC2700030 18929686

[CR31] Sobell L, Sobell M. Timeline follow-back: A technique for assessing self-reported alcohol consumption. Measuring Alcohol Consumption: Psychosocial and biochemical methods. Humana: NJ; 1992. pp. 41–72. L. RZ, A. JP, and T. NJ, Editors.

[CR32] Budney AJ, Novy PL, Hughes JR. Marijuana withdrawal among adults seeking treatment for marijuana dependence. Addiction. 1999;94(9):1311–22. 10615717 10.1046/j.1360-0443.1999.94913114.x

[CR33] Budney AJ, et al. The time course and significance of cannabis withdrawal. J Abnorm Psychol. 2003;112(3):393–402. 12943018 10.1037/0021-843x.112.3.393

[CR34] Buysse DJ, et al. The Pittsburgh Sleep Quality Index: a new instrument for psychiatric practice and research. Psychiatry Res. 1989;28(2):193–213. 2748771 10.1016/0165-1781(89)90047-4

[CR35] George SR, Clark M, Crotty M. Development of the Adelaide driving self-efficacy scale. Clin Rehabil. 2007;21:56–61. 17213242 10.1177/0269215506071284

[CR36] Fares A, et al. Combined effect of alcohol and cannabis on simulated driving. Psychopharmacology. 2022;239(5):1263–77. 33544195 10.1007/s00213-021-05773-3

[CR37] North AC, Hargreaves DJ. Music and driving game performance. Scand J Psychol. 1999;40:285–92.

[CR38] Di Ciano P, et al. Cannabis and Driving in Older Adults. JAMA Netw Open. 2024;7(1):e2352233. 38236599 10.1001/jamanetworkopen.2023.52233 PMC10797455

[CR39] Beal SL. Ways to fit a PK model with some data below the quantification limit. J Pharmacokinet Pharmacodyn. 2001;28(5):481–504. 11768292 10.1023/a:1012299115260

[CR40] Behzad D, et al. Effects of different methods of cannabis use on cognition and blood THC: A systematic review. Prog Neuropsychopharmacol Biol Psychiatry. 2025;139:111399. 40368229 10.1016/j.pnpbp.2025.111399

[CR41] Spindle TR, et al. Assessment of cognitive and psychomotor impairment, subjective effects, and blood THC concentrations following acute administration of oral and vaporized cannabis. J Psychopharmacol. 2021;35(7):786–803. 34049452 10.1177/02698811211021583 PMC9361180

[CR42] Zhao S, et al. The effect of cannabis edibles on driving and blood THC. J Cannabis Res. 2024;6(1):26. 38822413 10.1186/s42238-024-00234-y PMC11140993

[CR43] Won NY et al. Edible cannabis use on simulated driving performance. Traffic Inj Prev, 2025: pp. 1–9. 10.1080/15389588.2025.2574271 PMC12967284 41223382

[CR44] Bosker WM, et al. Medicinal Delta(9) -tetrahydrocannabinol (dronabinol) impairs on-the-road driving performance of occasional and heavy cannabis users but is not detected in Standard Field Sobriety Tests. Addiction. 2012;107(10):1837–44. 22553980 10.1111/j.1360-0443.2012.03928.x

[CR45] Miller R, et al. Influence of cannabis use history on the impact of acute cannabis smoking on simulated driving performance during a distraction task. Traffic Inj Prev. 2022;23(s1):S1–7. 35686998 10.1080/15389588.2022.2072492 PMC10108575

[CR46] Matheson J, et al. Sex differences in the acute effects of smoked cannabis: evidence from a human laboratory study of young adults. Psychopharmacology. 2019;237(2):305–16. 31637452 10.1007/s00213-019-05369-y

[CR47] Hartman RL, et al. Cannabis effects on driving longitudinal control with and without alcohol. J Appl Toxicol. 2016;36(11):1418–29. 26889769 10.1002/jat.3295

[CR48] Hartman RL, et al. Cannabis effects on driving lateral control with and without alcohol. Drug Alcohol Depend. 2015;154:25–37. 26144593 10.1016/j.drugalcdep.2015.06.015 PMC4536116

